# Examining Interpersonal Traumas Across Low Income Latinx Mother-Youth Dyads: Associations Between Maternal Child Abuse Exposure and Racial Discrimination with Mother and Youth Psychopathology

**DOI:** 10.1007/s10578-022-01483-9

**Published:** 2022-12-19

**Authors:** Lyric N. Russo, Jose Arreola, Gloria Montiel, Gina Torres, Francisca Leal, Nancy Guerra, Jessica L. Borelli

**Affiliations:** 1 University of California, 4201 Social and Behavioral Sciences Gateway, 92697 Irvine, CA United States; 2 Latino Health Access, Santa Ana, CA United States

**Keywords:** child abuse, racial discrimination, Latinx, mental health, mother-youth dyads, intergenerational impact

## Abstract

Child abuse has intergenerational consequences for psychopathology, however, there remains a paucity of research regarding how these experiences affect Latinx families, particularly those at risk for additional negative life events, such as racial discrimination. This study aims to contribute to this gap in the literature by examining the impact maternal child abuse exposure has on youth and maternal psychopathology, as well as whether these associations are moderated by racial discrimination, in a sample of 224 Latinx mother-youth dyads. Hierarchical regressions revealed small but significant *maternal child abuse exposure* x *racial discrimination* interactions for youth depression and anxiety, but not maternal depression or anxiety, which were solely positively associated with maternal child abuse exposure. Findings highlight the multifarious, and at times convergent, nature of trauma and oppression among Latinx families, as well as the impact across generations. Future work is needed to further elucidate developmental pathways of intergenerational trauma in understudied populations.

Child abuse (CA) is a widespread public health problem that refers to a range of child maltreatment experiences, including physical abuse, sexual abuse, and exposure to domestic violence during childhood [1]. In 2019, Child Protective Services (CPS) received approximately 4.4 million CA allegations for roughly 7.9 million children [2], however given that those numbers only reflect reported cases; it is estimated that 50–90% of children who experience abuse do not receive a formal report [3]. In fact, a recent examination of national surveys on children’s exposure to violence estimated that 38.1% of children had experienced neglect and abuse by the age of 17 [4], illustrating the pervasive nature of CA.

​​The public health toll of CA is clear --- exposure to CA is associated with a myriad of deleterious mental health consequences for survivors [5], including behavioral disorders [6], posttraumatic stress [7], and mood disorders [7], all of which can persist well into adulthood [8, 9]. Latinx individuals, who already must contend with systemic oppression [10], sociodemographic adversity [10], and racism and discrimination [11], are at heightened risk for CA due to elevated rates of family and community violence exposure, as well as decreased access to preventative resources and services [12, 13].

## Intergenerational Consequences of Child Abuse Exposure on Youth Psychopathology

In addition to the negative associations for the survivor, there are also intergenerational consequences of abuse exposure [14]. Although not CA per se, research suggests that parental trauma exposure is associated with child psychopathology and behavioral issues even in the absence of child trauma exposure [14]. Studies examining the intergenerational consequence of CA exposure have shown that, while there are exceptions and mixed findings [15], parental exposure to CA is generally associated with higher potential for CA perpetration [16], as well as worse psychopathology in offspring [17, 18].

Investigations of maternal CA exposure and psychopathological outcomes in all-Latinx samples is limited, although recent studies have begun exploring how trauma and adversity affects future generations of Latinx individuals [see 19 for a review]. Studies with majority Latinx samples (over 50% identifying as Latinx) have found that mothers with childhood trauma exposure (including childhood physical abuse) report significantly more socioemotional problems in their children [20] and that maternal CA exposure predicts child internalizing and externalizing disorders [21]. Most of these studies, however, have primarily examined younger children (ages 3 to 10) with parent-reported outcomes, thereby obscuring the experiences of older children who can report on their own symptomatology and may provide insight into how maternal CA exposure impacts youth mental health. Examining the experiences of older youth is particularly important in adolescence, as anxiety and depressive symptoms are shown to sharply increase during this time, particularly among girls [22], and such information has the potential to inform intervention avenues for this difficult to reach age group.

Efforts to understand the intergenerational toll of CA exposure indicate that, among such factors as heightened stress reactivity and epigenetic factors, parental CA exposure may affect children through insensitive parent-child interactions [23]. For example, a child’s behaviors (e.g., aggression) can serve as a trauma trigger for parents, and thus may elicit maladaptive responses from parents coping with traumatic experiences that are indicative of their trauma history [24]. Similarly, CA-exposed parents may exhibit behaviors consistent with symptomatology of depression and anxiety, such as irritation, persistent negative emotional states, detachment from others, or angry outbursts, which if directed towards or observed by the child can create a state of fear [25, 26] or encourage reenactment of such behavior [27]. Parents may also be unable to provide the necessary support to a child experiencing distress if they themselves are trying to cope with their own psychopathological symptoms [28].

## Risk Factors Heightening the Impact of Child Abuse Exposure on Psychopathology

In the aftermath of CA exposure, however, not everyone at risk experiences the same outcome [29]. Developmental psychopathology suggests that the level of adaptive functioning following an inciting event, such as CA exposure, is a result of the interactive effect between risk and protective factors [30]. This framework emphasizes the phenomena of equifinality and multifinality: different risk factors may result in the same outcome, and specific risk factors may result in a multitude of outcomes. Additional work, however, is needed to better understand the factors associated with multifinality in this particular developmental pathway, particularly within historically excluded and marginalized communities.

Racial discrimination (RD), or the unequal and unjust treatment of members of an ethnic or racial group on the basis of race [31], has received attention as a potential risk factor for psychopathology as exposure to RD is associated with worse psychopathological functioning in both youth and adults [32–34], as well as considered its own form of trauma [35]. A meta-analysis by Williams and colleagues (2003) suggested that the link between RD and psychopathology may be especially strong due to the impact discrimination has on one’s perception of themselves and their surroundings [36]. For instance, RD, which a majority of Latinx youth [37] and approximately 4 in 10 Latinx adults report experiencing in the past year [11], may lead to affective reactions, such as sadness, that shape one’s interpretation of their world and may impact one’s ability to adaptively respond to experiences by threatening one’s sense of control and fostering hopelessness [38]. Further, RD may trigger negative emotional states that influence both cognitive and biological heightened stress responses, that in turn place individuals at increased risk for mental health difficulties [39], as well as mimic the function of a traumatic stressor (i.e., higher levels of anxiety, guilt/shame, avoidance, and hypervigilance).

For mothers with a history of CA exposure, maternal RD may act as a stressor that retraumatizes the individual and leads to worse psychopathological outcomes [40]. Cicchetti and Lynch’s Ecological-Transactional model (1993), for instance, provides a lens with which to examine this interaction as this developmental framework moves beyond restricting events to the experience of the individual and aims to examine the more complex context of interacting systems, including family, community, and sociopolitical climate on mental health [41]. Building on Bronfenbrenner’s Ecological Systems Theory (1977), the Ecological-Transactional model places individuals at the center of their ecology, impacted by direct and indirect events throughout their lifespan, and purports that adverse experiences that impact children have a greater impact on them; for instance, exposure to CA has far-reaching effects on the person’s developing stress biology. By emphasizing the importance of stressors originating both inside and outside the family and how they may influence development, this framework has particular relevance for examining the relationship between early life stressors, such as maternal CA exposure, ongoing stressors, such as maternal RD exposure, and their interactive impact on maternal mental health outcomes in historically marginalized communities [34].

Youth exposed to RD are also at risk for negative outcomes, particularly when they do not have an outlet to discuss the discriminatino or if they do not have a parent who is able to provide emotional support/reassurance that such discrimination is not indicative of them but rather the person hailing the discrimination [42]. Mothers with a history of CA exposure, for example, may have difficulty recognizing their child’s needs due to preoccupation with their own anxiety or depressive symptomatology [28]. Additionally, CA exposure may distort a parent’s perceptions of their child’s needs and disrupt their ability to provide an attuned response to counteract the negative consequences associated with RD [23]. In other words, experiencing RD in addition to having a parent with a history of CA exposure may result in increased psychopathological stress for youth as they are not only experiencing interpersonal trauma (RD) but also lack an emotional outlet to understand and process the abuse. Given that exposure to RD increases in adolescence [42], examining these associations during middle childhood and adolescence may be particularly important.

Few studies, however, and none with an all-Latinx sample looking at CA exposure, have examined maternal RD as a moderator in relation to maternal trauma history. Of the few that have looked at interpersonal trauma, Mekawi and colleagues (2021) revealed a significant interaction between maternal RD and interpersonal trauma in predicting psychopathology, such that the association between posttraumatic stress symptoms and interpersonal trauma was stronger at higher levels of reported maternal RD in a sample of Black women [40]. Additional research is needed, however, to explore these associations and their impact on anxiety and depression, particularly in relation to maternal CA exposure, as both forms of trauma are extensively linked to increased anxiety and depressive symptoms in adults [7]. In terms of youth, no study to our knowledge has examined the interactive effects of maternal CA exposure and youth RD exposure on youth psychopathology, underscoring the importance of examining their interactive toll on Latinx youth anxiety and depression. By examining the dual impact of maternal CA exposure and youth RD exposure, we aim to investigate whether maternal trauma exposure (CA) and youth RD interact to predict increased youth anxiety and depressive symptoms in low-income Latinx youth. Understanding such processes has the potential to inform future interventions aimed at fostering psychological and relational health for Latinx youth and their families, as well as to identify intersectional risk factors.

## Current Study

This study aims to contribute to this gap in the literature by examining the intergenerational consequences maternal CA exposure has on child and maternal psychopathology, as well as whether these associations are moderated by maternal and youth RD exposure, in a sample of Latinx mothers and youth between the ages of 8 and 17. Studies reveal similarities in mental health outcomes in children and youth following trauma exposure [43, 44] and meta-analyses have failed to find age-related differences in the association between trauma and youth mental health [45], justifying use of this age range.

To accomplish these aims, we assessed youth and mothers’ anxiety and depressive symptoms, as well as their exposure to RD. Mothers also reported on their history of CA exposure and demographic information. Maternal CA exposure was assessed as a single variable, rather than by form of abuse, as extant literature has illuminated that Latinx individuals are at elevated risk for polyvictimization (e.g., victimization across various domains of trauma, including sexual and physical abuse) compared to white individuals [46, 47], yet limited research has examined the collective toll of CA exposure within these populations.

We tested three hypotheses. First, we predicted that maternal CA exposure would be associated with greater psychopathological symptoms for youth and mothers (Hypothesis 1). Second, we predicted that youth/maternal RD exposure would be associated with greater psychopathological symptoms for youth and mothers (Hypothesis 2). Finally, we predicted that youth/maternal RD would moderate the association between maternal CA exposure and psychopathology for youth and mothers (Hypothesis 3), such that the positive association between maternal CA exposure and psychopathology would increase in the presence of reported youth/maternal RD exposure.

## Method

This study was approved by the PI’s Institutional Review Board (HS# 2017–3974). The study sample was drawn from a collaborative community intervention study and includes both control and intervention mother-youth dyads from their baseline assessments. Promotoras (trained community workers) at a community health agency (Latino Health Access) located in an urban inner city serving low income neighborhoods conducted outreach to recruit families to participate in a family wellness program offered through a university partnership with the community health agency. Eligibility included having a child in the desired age range, being free of developmental disability or severe mental illness (e.g., psychosis), per mothers’ report, and residing in one of the target neighborhoods selected by the researchers due to elevated risk for poverty and exposure to violence (see 48). We began the study engaging youth between the ages of 11 and 17 but expanded the range to include youth between the ages of 8 and 17 at the promotoras’ suggestion that the current political climate was making families wary to participate in research, necessitating that we broaden inclusion criteria.

The resultant sample consisted of *N* = 224 mothers (*M*_age_= 40.86, *SD*_age_= 6.58) and youth (*M*_age_= 12.24, *SD*_age_= 2.09; 48.2% female) dyads. On average, the mothers in the sample had 3 children (including the child participating in the study, *SD* = 1.10) and had completed eighth grade (*SD* = 2.99). The majority (95.3%) of mothers reported speaking Spanish most of the time, and 97.2% of the mothers reported they had not been born in the United States: 90.7% born in Mexico, and the remainder from El Salvador (2.7%) and Guatemala (1.3%). The majority of youth (94.8%) were born in the United States and reported speaking both Spanish and English at home (62.9%). The average annual income per household for the sample was $28,912.30–well under the census bureau’s poverty guideline for a family of five (the mean family size for this sample) residing in California ($31,040.00; https//aspe.hhs.gov/poverty-guidelines 2021).

### Procedure

Eligible participants were provided with additional information regarding the study by trained bilingual research assistants. Interested mothers and youth provided informed consent and assent before proceeding with the assessment where they were given paper and pencil questionnaires in their preferred language (Spanish or English) to complete. All mothers completed assessments in Spanish, while youth completed assessments in English. Families were randomized to an intervention or waitlist control group.

### Measures

**Child abuse exposure**. Mothers completed the Traumatic Life Events Questionnaire (TLEQ) [49], a 23-item scale measuring whether an individual has experienced a wide range of traumatic events, including CA exposure. Five items meet the criteria for CA: one focusing on physical abuse, one focusing on domestic violence, and three focusing on child sexual abuse. Mothers’ victimization history was calculated continuously (0 to 3) based on the form of abuse. This type of scoring has been utilized extensively in ACEs literature and this measure has previously been used to identify CA within adult samples [50].

**Racial discrimination.** Youth and mothers each reported on RD by completing 1-item (e.g., *How often do people treat you unfairly because of your ethnicity/race?*) from the Perceived Discrimination Scale [51], a 3-item scale assessing discrimination in daily life developed for Latinx individuals living in the United States. The single item was selected for measurement as the other two items assessed the respondents’ perception of friends being discriminated against, which does not capture their own experience, as well as how “disliked” they felt by others. Respondents answered the question on a 4-point scale from 1 (*Never*) to 4 (*Always*). As with our CA variable, we assessed youth and maternal RD exposure continuously. This 1-item scale has previously been used to identify RD exposure within Latinx adult samples [52].

#### Youth Psychopathology

As discussed above, eligibility for this investigation initially involved youth ages 11–17. However, due to challenges with recruitment, part-way through the data collection phase, we extended the age range of eligible youth to 8 to 17 year-olds. This resulted in measures which had previously been selected for the initial age range to not be appropriate for younger ages (8–10 years). Thus, we selected additional measures that were appropriate for the younger age range. To address the fact that we had different measures, we created standardized *z*-scores to place all measures on the same scale for analysis. Support for the validity of this approach exists in the form of their association with parent-child relationship quality and other indices of mental health [48, 53]. 

**Anxiety.** Children (*n* = 47, ages 8–10) reported on their anxiety symptoms using the Multidimensional Anxiety Scale for Children (MASC) [54], a 39-item questionnaire that presents youth with a series of statements (e.g., *The idea of going away to camp scares me*) and asks them to decide how true each statement is for them on a 4‐point scale from 0 (*Never*) to 3 (*Often*). This measure has high validity and reliability in past studies with clinical and nonclinical populations [54], as well as with Latinx youth samples [55]. Cronbach’s ɑlpha was good, α = 0.83.

Adolescents (*n* = 177, ages 11–17) reported on their anxiety symptoms using the Youth Self Report [YSR; 57], which assesses broadband psychopathology among youth ages 11–18. This study utilized the anxiety problems subscale (9 items, α = 0.74; e.g., *I’m afraid of going to school*). Youth rated each item on a 3-point scale from 0 (*Not True*) to 2 (*Very True* or *Often True*). The YSR has previously been utilized with Latinx youth samples [57].

**Depression**. Children (*n* = 47, ages 8–10) reported on their depression symptoms using the Child Depression inventory (CDI) [58], a 27-item assessment (α = 0.85) that taps into the cognitive, emotional, behavioral, and psychological aspects of depression. Participants are presented with a series of three statements and are asked to select the statement that fits their experience over the past two-weeks the best (e.g., *I am sad once in a while, I am sad many times, or I am sad at all times*). Higher scores indicate more severe depression. The psychometric properties of the CDI are strong [58] and has previously been used with Latinx youth samples [59].

Adolescents (*n* = 177, ages 11–17) reported on their own depressive symptoms using the YSR [56]. This study utilized the depression problems scale (13 items, α = 0.82; e.g., *I feel bad that no one loves me*). Youth rated each item on a 3-point scale from 0 (*Not True*) to 2 (*Very True* or *Often True*).

#### Maternal Psychopathology

**Anxiety.** Mothers reported on their own anxiety using the 6-item (α = 0.88) subscale of the 18-item Brief Symptom Inventory (BSI-18) [60]. Items assess anxious feelings (e.g., *During the past week including today, how much were you distressed by nervousness or shakiness inside?*) and are rated on a 5-point Likert scale from 0 (*Not at all*) to 4 (*Extremely*). Past studies have illustrated good reliability and validity for low-income Latinx mothers [61].

**Depression**. Mothers reported on their own depressive symptoms using the 6-item (α = 0.86) subscale of the 18-item BSI [60]. Items (e.g., *During the past week, how much were you distressed by feelings of worthlessness?*) are rated on a 5-point Likert scale from 0 (*Not at all*) to 4 (*Extremely*).

### Measure Validation for Spanish-Speaking Latinx Mothers

Of the measures used in this study, the TLEQ and the Perceived Discrimination Scale had not previously been translated and validated in Spanish. To address this issue, before administering these measures, we conducted an online validation study using an independent sample of *N* = 215 Spanish-speaking Latinx mothers residing in the United States. Each measure was translated into Spanish using the forward-back translation method to ensure accuracy. Participants were recruited through email to social networks (*n* = 205) and Mechanical Turk (*n* = 10). To validate the TLEQ, we selected our convergent measure to be the Spanish adaptation of the Traumatic History Questionnaire (THQ) [62] as it has been used to establish validity with other native Spanish-speaking samples. The Spanish version of the THQ is a 24-item self-report instrument designed to assess an individual’s trauma and abuse history. The Spanish version of the Self-Compassion Scale (SCS) [63], a self-report measure assessing mindfulness, was utilized as a measure of divergent validity. To validate the Perceived Discrimination Scale we selected the discrimination subscale of the Hispanic Stress Inventory Version 2 (HSI‐V2) [64], a self‐report assessment identifying discrimination experiences in immigrant samples, as our convergent measure. The Spanish version of the Multidimensional State Boredom Scale (MSBS) [65] was selected as the divergent measure of validity. Tables 1 and 2 in the appendix display the significant findings that validate our Spanish versions of the TLEQ and the Perceived Discrimination Scale.

**Table 1:** Bivariate correlations and descriptive statistics among key study variables

	Y Age	Y Sex	M Age	M Edu	Income	M CA	Y RD	M RD	Y Anx	Y Dep	M Anx	M Dep
*M(SD)*	*12.25(0.56)*	*1.52(0.56)*	*40.86(6.58)*	*8.65(2.99)*	*$28,912(52,481)*	*0.92(1.08)*	*1.31(0.61)*	*1.37(0.54)*	*0.00(1.00)*	*0.00(1.00)*	*2.65(3.56)*	*3.31(3.91)*
**Alphas**	**-**				**-**	**-**	**-**	**-**	**α = .84a, .72b**	**α = .82a, .81b**	**α = 0.88**	**α = 0.86**
Y Age	1.00											
Y Sex	0.07	1.00										
M Age	0.10	0.10	1.00									
M Edu	-0.04	0.06	-0.11	1.00								
Income	0.04	0.07	0.07	0.05	1.00							
M CA	-0.09	0.06	-0.14*	0.10	-0.01	1.00						
Y RD	-0.20**	-0.17*	-0.14*	-0.06	-0.03	0.01	1.00					
M RD	-0.10	0.03	0.00	0.11	-0.06	0.11	0.06	1.00				
Y Anx	-0.05	0.06	-0.06	0.06	0.03	0.08	0.09	0.11	1.00			
Y Dep	-0.04	-0.01	0.06	-0.05	0.03	0.15*	0.24**	0.16*	0.53**	1.00		
M Anx	0.01	0.01	-0.02	0.01	-0.04	0.16*	-0.05	0.17*	0.07	0.06	1.00	
M Dep	0.09	-0.03	0.08	-0.02	-0.06	0.13	-0.04	0.10	0.11	0.16*	0.69**	1.00

Note: * *p* < .05, ** *p* < .01; *M(S*D) = mean and standard deviation; Alphas = Cronbach’s alpha; Y Age = age of youth in years; Y Sex = youth sex (1 = male, 2 = female); M Age = age of mothers in years; M Edu = mother’s highest completed level of education; Income = annual household income reported by mothers; M CA = maternal child abuse exposure reported via the TLEQ (continuous 0 to 3); Y RD = youth reported experiences of racial discrimination on the Perceived Discrimination Scale (continuous 0 to 4); M RD = mothers reported experiences of racial discrimination on the Perceived Discrimination Scale (continuous 0 to 4); Y Anx = standardized anxiety scores for the sample (for children, this is their score on the MASC, and for adolescents, this is their anxiety problems score on the YSR); Y Dep = standardized depression scores for the sample (for children, these are their scores on the Child Depression Inventory, and for adolescents, this is their depressive problems subscale score on the YSR); M Anx = anxiety symptoms on BSI; M Dep = depressive symptoms on BSI.

**Table 2:** Regression examining the main effects of maternal child abuse exposure and youth experiences of racial discrimination, as well as the interaction between the two in predicting youth psychopathology

	Y Anxiety			Y Depression		
	*b*/ΔR^2^	*SE*	95% CI	*b*/ΔR^2^	*SE*	95% CI
Step 1 ΔR^2^	0.01			0.01		
Y Age	-0.02	0.04	[-0.09, 0.05]	-0.02	0.04	[-0.09, 0.05]
Y Sex	0.09	0.13	[-0.16, 0.35]	-0.03	0.13	[-0.29, 0.23]
M Age	-0.01	0.01	[-0.03, 0.01]	0.01	0.01	[-0.01, 0.03]
M Edu	0.01	0.02	[-0.04, 0.06]	-0.02	0.03	[-0.07, 0.03]
Income	-0.14	0.16	[-0.46, 0.18]	-0.26	0.16	[-0.59, 0.07]
Condition	-0.02	0.07	[-0.17, 0.13]	0.02	0.08	[-0.13, 0.17]
Step 2 ΔR^2^	0.00			0.06**		
M CA	0.01	0.07	[-0.13, 0.15]	0.15*	0.07	[0.02, 0.29]
Y RD	0.02	0.13	[-0.24, 0.28]	0.39**	0.13	[0.14, 0.65]
Step 3 ΔR^2^	0.02*			0.03*		
M CA x Y RD	0.24*	0.12	[0.01, 0.48]	0.29*	0.12	[0.06, 0.53]

Note: * *p* < .05, ** *p* < .01; Y Age = youth’s age; Y Sex = youth sex (1 = male, 2 = female); M Age = age of mothers in years; M Edu = mother’s highest completed level of education; Income = annual household income reported by mothers; Intervention Condition = participant assignment to intervention or control condition; M CA = maternal child abuse exposure reported via the TLEQ (continuous 0 to 3); Y RD = youth reported experiences of racial discrimination on the Perceived Discrimination Scale (continuous 0 to 4); M CA x Y RD = interaction between maternal child abuse exposure and youth experience of racial discrimination.

### Data Preparation

As described above, to accommodate our use of age-appropriate measures (e.g., child anxiety was measured with the MASC at ages 8–10 and the YSR for ages 11–17), we standardized youth’s scores within each measure and used these z-scores as our measures of the construct of interest (e.g., anxiety). This procedure was used for each of the constructs assessed with different scales for specific age ranges (i.e., YSR-depression and CDI, YSR-anxiety and MASC).

### Data Analyses

To test study hypotheses, we conducted hierarchical linear regressions. In the first step of these regressions, we controlled for youth age/sex, maternal age, annual household income, maternal education, and intervention condition, as past research has illustrated that these factors are associated with trauma-related psychopathological outcomes [66, 67]. On the second step, we entered the main independent variables of interest on psychopathology: maternal CA exposure and (youth or mother reported) RD exposure (Hypotheses 1 and 2). On the third step, we entered the interaction between maternal CA exposure and (youth or maternal) RD, which allowed us to test for Hypotheses 3 (interaction between maternal CA exposure and RD exposure in the prediction of youth and mother psychopathology). Hayes’ PROCESS macro was utilized to probe the simple slopes from the moderation analyses [68].

## Results

Nearly half (49.6%) of mothers in our sample reported a history of CA exposure, while 50.4% of mothers and 25.1% of youth reported experiencing RD. Of those with a history of CA exposure, witnessing domestic violence was the most endorsed experience (36.6%), followed by sexual abuse (32.1%) and physical abuse (20.5%), with most participants (54.1%) endorsing at least two categories of CA. Approximately 13% (n = 29) of mothers reported elevated anxiety symptoms, and 19% (n = 43) of mothers reported elevated depressive symptoms (i.e., met the clinical threshold for concern on the BSI-18). Approximately 13% (n = 29) of mothers reported elevated anxiety symptoms, and 19% (n = 43) of mothers reported elevated depressive symptoms (i.e., met the clinical threshold for concern on the BSI-18). On average, depressive symptoms on the BSI were comparable to rates from other low-income Latinx samples in the United States (*M*_dep_ = 2.65, *SD* = 3.56 compared to *M*_dep_ = 2.54, *SD* = 5.08, [61]), however, t-tests revealed that mothers in our sample reported significantly higher anxiety symptoms (M_anx_ = 3.31, *SD* = 3.91 compared to M_an_ = 2.33, *SD* = 4.82, [61]) compared to similar Latinx samples (*p* = .01), although this may be due to the time frame in which our data were collected (an era of higher sociopolitical concerns, especially among our sample, which included many participants with fears regarding deportation). Bivariate correlations revealed that youth age (*p* = .003), youth sex (*p* = .01), and maternal age (*p* = .046) were negatively correlated with youth RD exposure, while youth depression was positively correlated with youth RD exposure (*p* = .001), maternal RD exposure (*p* = .02), and maternal depression (*p* = .02). See Table 1 for bivariate correlations and descriptive statistics among key study variables.

### Hypothesis Testing

We examined the main effects of maternal CA exposure and youth/mother RD exposure in predicting youth and maternal psychopathology, as well as whether youth/maternal RD moderated the association between maternal CA victimization history and psychopathology, among mothers and youth.

#### Prediction of Youth Psychopathology

**Anxiety.** In an initial regression controlling for covariates (youth sex, youth age, maternal age, maternal education, annual household income, intervention condition), *R*^*2*^ = 0.01, *p* = .91, the step containing the main effects did not significantly contribute to the prediction of youth-reported anxiety, Δ*R*^*2*^ = 0.00, *p* = .98. Neither maternal CA exposure nor youth RD were significantly associated with youth anxiety (Hypotheses 1 and 2). However, the interaction in Step 3 contributed significant variance, Δ*R*^*2*^ = 0.03, *p* = .045 (Hypothesis 3; refer to Table 2 for regressions related to the prediction of youth psychopathology).

Simple slope tests revealed that the interaction effect was not significant at low (*p* = .43), moderate (*p* = .87), or high (*p* = .13) conditions. The Johnson-Neyman technique was then used to identify at what level of the moderator (RD exposure) the moderation became statistically significant revealing that the interaction significantly predicted an increase in anxiety for youth reporting > 3.4 on RD exposure (approximately 2% of our sample).

​​**Depression.** In a similar regression model examining depression, *R*^*2*^ = 0.01, *p* = .92, the step containing the main effects significantly contributed to the prediction of youth-reported depression, Δ*R*^*2*^ = 0.06, *p* = .001. Both maternal CA exposure, *b* = 0.15, *SE* = 0.07, *p* = .03, and youth RD exposure, *b* = 0.39, *SE* = 0.13, *p* = .003, were significantly positively associated with youth depression (Hypotheses 1 and 2). Similarly, the interaction contributed significant variance, Δ*R*^*2*^ = 0.03, *p* = .01 (Hypothesis 3).

**Fig. 1:**
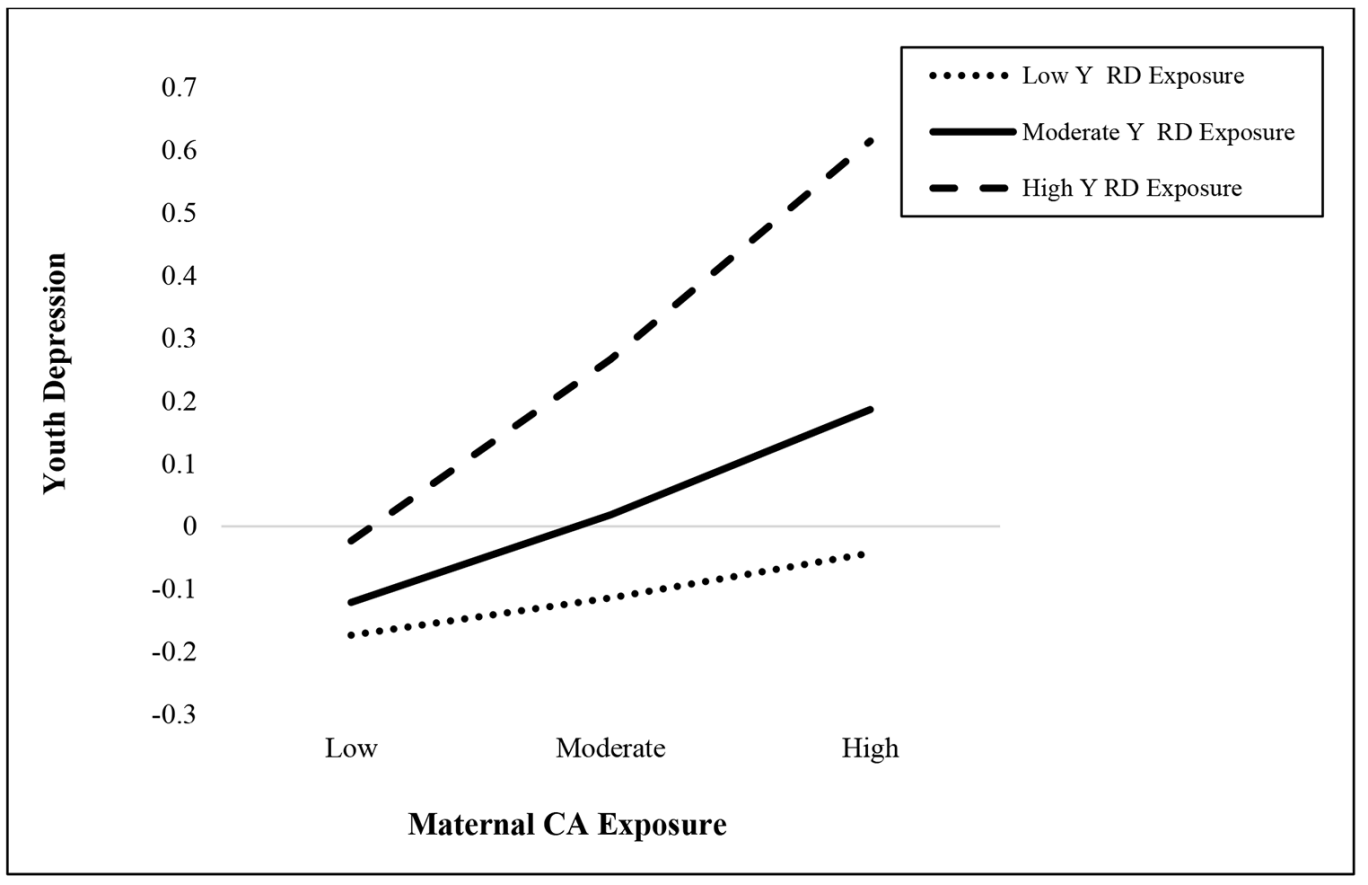
Interaction between maternal child abuse exposure and youth experiences of racial discrimination in the prediction of youth-reported depression. (Note: Youth Depression = standardized depression scores for the youth in this sample (for children, these are their scores on the Child Depression Inventory, and for adolescents, this is their depressive problems subscale score on the YSR); Maternal CA Exposure = maternal child abuse exposure reported via the TLEQ (continuous 0 to 3); Y RD Exposure = youth reported experiences of racial discrimination on the Perceived Discrimination Scale (continuous 0 to 4). Moderation analyses controlled for youth age, youth sex, maternal age, maternal education, income, and intervention condition)

Simple slope tests revealed a significant positive association between maternal CA exposure and youth depression among youth reporting moderate (*b* = 0.16, *SE* = 0.07, *p* = .03) or high (*b* = 0.33, *SE* = 0.10, *p* = .001) levels of RD but not among youth reporting low levels of RD (*b* = 0.07, *SE* = 0.08, *p* = .39), illustrating that youth depression was higher among youth whose mothers experienced CA exposure (refer to Fig. 1).

#### Prediction of Maternal Psychopathology

**Anxiety.** In an initial regression controlling for covariates (youth sex, youth age, maternal age, maternal education, annual household income, intervention condition), *R*^*2*^ = 0.00, *p* = .99, the step containing the main effects was not a significant predictor of maternal anxiety, Δ*R*^*2*^ = 0.03, *p* = .06. Only maternal CA exposure, *b* = 0.08, *SE* = 0.04, *p* = .03, not maternal RD exposure, was significantly associated with maternal anxiety (Hypotheses 1 and 2). The interaction did not contribute significant variance (Hypothesis 3; refer to Table 3 for regressions related to the prediction of maternal psychopathology).

**Table 3:** Regression examining the main effects of maternal child abuse exposure and maternal experiences of racial discrimination, as well as the interaction between the two in predicting maternal psychopathology

	M Anxiety			M Depression		
	*b*/ΔR^2^	*SE*	95% CI	*b*/ΔR^2^	*SE*	95% CI
Step 1 ΔR^2^	0.00			0.01		
Y Age	-0.01	0.02	[-0.05, 0.03]	0.01	0.02	[-0.03, 0.06]
Y Sex	0.00	0.07	[-0.14, 0.14]	-0.04	0.08	[-0.20, 0.13]
M Age	0.02	0.01	[-0.01, 0.05]	0.01	0.01	[-0.01, 0.02]
M Edu	0.02	0.01	[-0.03, 0.04]	-0.01	0.02	[-0.04, 0.02]
Income	0.10	0.09	[-0.08, 0.28]	0.05	0.11	[-0.16, 0.25]
Condition	0.00	0.04	[-0.09, 0.08]	-0.01	0.05	[-0.11, 0.08]
Step 2 ΔR^2^	0.03			0.02		
M CA	0.08*	0.04	[-0.01, 0.16]	0.09*	0.04	[0.00, 0.18]
M RD	0.08	0.08	[-0.08, 0.24]	0.06	0.09	[-0.13, 0.24]
Step 3 ΔR^2^	0.01					
M CA x M RD	-0.11	0.08	[-0.26, 0.04]	-0.10	0.09	[-0.28, 0.07]

Note: * *p* < .05, ** *p* < .01; Y Age = youth’s age; Y Sex = youth sex (1 = male, 2 = female); M Age = age of mothers in years; M Edu = mother’s highest completed level of education; Income = annual household income reported by mothers; Intervention Condition = participant assignment to intervention or control condition; M CA = maternal child abuse exposure reported via the TLEQ (continuous 0 to 3); M RD = mothers reported experiences of racial discrimination on the Perceived Discrimination Scale (continuous 0 to 4); M CA x M RD = interaction between maternal child abuse exposure and mothers experience of racial discrimination.

**Depression.** In a similar regression model controlling for covariates, *R*^*2*^ = 0.01, *p* = .87, the step containing the main effects did not significantly contribute to the prediction of maternal depression, Δ*R*^*2*^ = 0.02, *p* = .10. Only maternal CA exposure, *b* = 0.09, *SE* = 0.04, *p* = .04, not maternal RD exposure, was significantly associated with maternal depression (Hypotheses 1 and 2). Similarly, the interaction did not contribute significant variance (Hypothesis 3).

## Discussion

This study aimed to evaluate the individual and interactive associations of maternal CA exposure and youth/maternal RD exposure with youth and maternal psychopathology in a sample of Latinx mother-youth dyads. Although Latinx individuals represent the largest growing minority group in the United States [69], one with heightened levels of CA exposure [70], discrimination [11], and untreated mental health concerns [71], few studies have examined the impact maternal CA exposure has on psychopathology in this population. Additionally, no studies to our knowledge have examined youth/maternal RD as a risk factor for youth and mother psychopathology following maternal CA exposure, despite extensive evidence linking both CA and RD to worsening mental health outcomes [7, 72]. Thus, this study constitutes an important contribution to the literature by offering an in-depth assessment of psychopathology and risk factors within a sample of low-income Latinx youth and mothers.

In terms of the incidence within our sample, it is worth noting that mothers reported rates of CA exposure (49.6%) comparable to national lifetime estimates (38.1%)[4]. Of those who reported CA exposure, witnessing domestic violence was the most frequently endorsed experience, followed by sexual abuse, and physical abuse, with most participants reporting at least two types of CA exposure. These findings align with national prevalence rates of traumatic events [73], although our sample reported slightly higher rates for all forms of abuse, a finding on par with other Latinx community samples [74]. Similarly, approximately half (50.4%) of the mothers reported RD, a figure which aligns with national estimates for Latinx adults in the U.S. [11]. For youth, RD exposure was reported at a lower rate than other Latinx community samples (25% compared to 50–80%; 38, 43].

We first hypothesized that maternal CA exposure, in isolation, would be associated with higher levels of psychopathology for both youth and their mothers. This hypothesis was largely supported. Specifically, maternal CA exposure alone significantly predicted youth depression, maternal anxiety, and maternal depression, but not youth anxiety. Our findings corroborate that of previous research in identifying a significant positive association between maternal trauma history and both youth and maternal depression as well as maternal anxiety [5, 7]. As expected, youth of mothers with a history of CA exposure reported increased depressive symptoms compared to youth of mothers not reporting CA exposure. One untested explanation for this pattern of effects is that mothers who have more extensive victimization histories engage in more insensitive parenting, which may impede youth’s feelings of safety or confidence in their surroundings, and thus how they view themselves and the world around them [23]. Child abuse exposure has also been shown to be associated with more frightened and dissociated parenting behaviors [25], which, if directed or observed by the child, can invoke fear in the child and place them in a paradox that feels as if the parent is directly threatening them [25]. Alternatively, it could be that the youth of mothers with a history of CA exposure are exposed to additional risk factors (such as higher levels of community violence or socioeconomic stress) that are responsible for their higher depressive symptoms. We attempted to account for this by statistically controlling for household income and education, but we did not measure community violence exposure and it is possible that there are other confounding factors that separate the families in which mothers had more extensive CA exposure from those who had less.

On the other hand, when examined in isolation, maternal CA exposure did not significantly predict youth anxiety. While unexpected, this finding is consistent with prior literature that has also failed to find a significant association between lifetime Latinx maternal trauma exposure and youth anxiety [19]. To contextualize our findings, it may be important to consider the clinical severity of the sample – children aged 8 to 10 in our sample reported higher anxiety on the MASC (*M* = 84.41) compared to other Latinx community samples (*M* = 62.53) [75], while adolescents aged 11 to 17 reported slightly lower anxiety on the YSR (*M* = 3.72) compared to similar Latinx samples (*M* = 5.16) [76]. Given that we used different measures for the different age groups in our sample, it is difficult to ascertain if the observed differences are due to measurement differences or to true differences as a function of participant age. Our sample was underpowered to examine the association between maternal CA and child anxiety separately within the age groupings, but examination of the beta weights indicates that there was not a significant difference between the two groups. These results highlight the need for continued examination of risk and protective factors within this population to elucidate the impact maternal CA victimization history has on mother and youth psychopathology. Such findings have the potential to inform intervention and prevention efforts for Latinx survivors of CA, as well as their families.

Hypothesis 2 held that RD exposure alone would be associated with higher levels of psychopathology for youth and their mothers. This prediction was largely unsupported, conflicting with prior research [32–34]. In fact, in isolation, RD exposure was only significantly associated with youth depression, failing to predict youth anxiety, maternal depression, and maternal anxiety on its own. Here, it is important to consider that Latinx individuals are at heightened risk for poly-victimization (e.g., victimization across various domains of trauma, including sexual, physical, and community violence) [46, 47], as well as an array of socioecological stressors [10], all of which may lead to desensitization to certain forms of trauma [77], particularly forms of trauma that are persistent and systemic, such as RD, resulting in decreased psychophysiological reactivity to such stress. This pattern may be particularly impactful when taking into consideration the additional stressors, such as CA exposure, for which nearly half the mothers in our sample experienced. In terms of youth depression, our findings reveal that youth RD exposure was associated with worse depressive symptoms. This is likely due to the toll RD exposure has on youth’s perception of themselves and their surroundings [36], as well as RD exposure’s ability to trigger negative emotional states that influence both cognitive and biological heightened stress responses [39] and mimic ​​the function of a traumatic stressor [38], likely resulting in worse depressive symptoms. Future research should explore distinct aspects of discrimination experiences for Latinx youth and adults, such as type and duration, to improve knowledge of causal pathways and to determine the relationship between RD and psychopathology in this population.

Finally, we had hypothesized that RD exposure would moderate the relationship between maternal CA exposure and psychopathology in youth and mothers, such that the positive association between maternal CA exposure and psychopathology would increase in the presence of youth or maternal RD exposure. This hypothesis was partially supported. The interaction between youth RD exposure and maternal CA exposure was significant in predicting youth anxiety and depression, but the interaction between maternal RD exposure and maternal CA exposure did not predict maternal anxiety or depression.

Our analyses revealed that only at very high levels of youth RD exposure (> 3.4 out of 4) were maternal CA exposure and anxiety positively associated, indicating that, while maternal CA exposure alone may not influence anxiety, when combined with very high levels of youth RD exposure, maternal CA exposure predicts an increase in youth anxiety. In contrast, conditional analyses revealed that maternal CA exposure and youth depressive symptoms were only associated when youth RD exposure was at mean or higher levels. This suggests that maternal CA exposure predicts higher youth anxiety only when youth RD is extremely high, and maternal CA exposure predicts higher depression in Latinx youth when youth RD exposure is at mean or higher levels. Conceptually, this makes sense as youth with moms who have been mistreated (CA exposure) and who also experience mistreatment (RD) may be more likely to experience anxiety and depression due to the cumulative effect of mistreatment, which likely impacts the way in which one views the world as either harmful or hopeful. This is especially visible when considering youth’s depressive symptoms, as youth with mothers who experienced CA exposure but they themselves did not experience RD reported lower depressive symptoms, perhaps due to viewing the world in a less dangerous light compared to youth who experienced RD and had mothers who experienced CA exposure.

For mothers, the findings indicated that only maternal CA exposure was uniquely associated with mental health, whereas maternal RD exposure was not. This suggests that CA exposure has long-lasting implications for survivors and may overshadow other forms of adversity, such as RD, in impacting Latinx maternal psychopathology. These findings align with the Ecological-Transactional framework [41], which posits that the earlier a person experiences trauma then the larger impact it will have on their stress reaction across the lifespan. In other words, experiencing RD may not be traumatic enough to impact one’s psychopathology when they are already dealing with the aftermath of CA exposure.

### Strengths & Limitations

As the first examination of these research questions within an all-Latinx sample, this study represents an important contribution to the literature. We utilized measures that had all been previously validated with this population or we validated them with this population ourselves before conducting the study, enhancing the confidence we can derive from the conclusions drawn from this study. Additionally, youth reported on their own mental health, a rarity within intergenerational trauma literature for older youth. Finally, this study was conducted in collaboration with our community partner—recruitment efforts were led by promotoras and assessments were completed at the agency. This partnership allowed participants to experience a level of trust with the research staff and research process that may have not been possible without the university-community partnership, therefore enhancing the integrity of the data we obtained.

This study also carried some limitations. First, it was cross-sectional and correlational, thus, restricting our ability to infer causal inferences. The sample was drawn from the baseline assessment of a non-clinical community intervention in which families were recruited from neighborhoods identified as having high levels of inequalities. These specific qualifications limit the generalizability and implications of these results to non-Latinx, low-income populations, as well as those who may be unlikely to participate in an intervention (which may be those most in need of support and experiencing worse psychopathology). Although our theoretical model holds implications for the intergenerational consequences of CA exposure, we assessed mothers’ retrospective recall of CA, which can be influenced by recall biases. Additionally, our CA measure, derived from the TLEQ, did not capture instances of psychological abuse or neglect, nor specific characteristics of maternal CA exposure (age at exposure, chronicity, relation to perpetrator), thus limiting the ability to explore differences in the associations between maternal CA exposure and psychopathology for youth and mothers based on these influential factors. Similarly, our RD variable did not capture specific characteristics of discrimination exposure, such as type and durtion. Future research examining the moderating role of RD should include a more comprehensive assessment, taking into consideration both overt acts and microaggressions, as well as the frequency of such experiences. Finally, although the focus on Latinx families is a valuable contribution to the existing literature, generalizing to other minority groups may be limited due to differences in discrimination experiences, underscoring the need for additional research.

Additionally, when interpreting these results, it is important to note that the main effects from this study were relatively small and accounted for only 2–6% of the variance in the model, while the interaction effects accounted for an additional 2–3%. From a developmental psychopathology perspective [30], these findings may reveal a small predictive pathway towards risk for Latinx youth and their mothers. Additional research, however, is needed to isolate, identify, and investigate other relevant factors (e.g., parent-child relationship dynamics, maternal posttraumatic stress symptomatology, youth CA exposure, community violence) that further contribute to youth and adult psychopathological outcomes when experienced in conjunction with maternal CA exposure and individual exposure to RD, particularly in longitudinal samples.

## Summary

Child abuse victimization history has intergenerational consequences for psychopathology, however, few studies examine the ways in which these experiences affect Latinx families, particularly those at risk for additional insults, such as RD. This study aimed to contribute to this gap in the literature by examining the impact maternal child abuse victimization history has on youth and maternal psychopathology, as well as whether these associations were moderated by youth or maternal racial discrimination exposure. Participants were 224 Latinx mothers (*M*age = 40.86, *SD*age = 6.58) and youth (*M*age = 12.24, *SD*age = 2.09) dyads who participated in a community intervention. Results of hierarchical linear regressions support the argument that maternal CA victimization history could constitute an intergenerational risk factor for the development of psychopathology in Latinx youth, underscoring the importance of screening for parents’ abuse history in prevention and treatment settings. Further, youth who experienced RD and had mothers with CA victimization reported higher anxiety and depressive symptoms, highlighting how multiple forms of oppression may lead to worsening psychopathological outcomes in adolescence. Thus, when providing treatment or intervention services to families with a history of CA, it may be important to consider modifications to help parents and youth identify additional stressors, such as RD, to build effective coping mechanisms and parenting practices. For mothers, our findings illustrate that only CA victimization history was associated with worse anxiety and depressive symptoms. Altogether, our findings highlight the multifarious, and at times convergent, nature of abuse and oppression among Latinx families, as well as the impact across generations. These results underscore the importance of culturally-informed family interventions that mitigate the adverse effects of trauma on parenting and mental health, as well as echo the need for increased investments in mental health resources and services in communities that have long been marginalized due to racial discrimination.

## References

[1] Legano L, Mchugh MT, Palusci VJ (2009) Child abuse and neglect. Curr Probl Pediatr Adolesc Health Care 39:1–26 10.1016/j.cppeds.2008.11.001 19138647

[2] U.S. Department of Health & Human Services (U.S. DHHS) Children’s Bureau (2021). Child maltreatment 2019. https://www.acf.hhs.gov/cb/research-data-technology/statistics-research/child-maltreatment

[3] Leeb RT, Lewis T, Zolotor AJ (2011) A review of physical and mental health consequences of child neglect and implications for practice. Am J Lifestyle Med 5:454–468 10.1177/1559827611410266

[4] Finkelhor D, Turner HA, Shattuck A, Hamby SL (2015) Prevalence of childhood exposure to violence, crime, and abuse: results from the National Survey of Children’s exposure to violence. JAMA Pediatr 169:746–754 26121291 10.1001/jamapediatrics.2015.0676

[5] Springer KW, Sheridan J, Kuo D, Carnes M (2007) Long-term physical and mental health consequences of childhood physical abuse: results from a large population-based sample of men and women. Child Abuse Negl 31:517–530 17532465 10.1016/j.chiabu.2007.01.003 PMC3031095

[6] Hazen AL, Connelly CD, Roesch SC, Hough RL, Landsverk JA (2009) Child maltreatment profiles and adjustment problems in high-risk adolescents. J Interpers Violence 24:361–378 18391059 10.1177/0886260508316476

[7] Higgins DJ, McCabe MP (2003) Maltreatment and family dysfunction in childhood and the subsequent adjustment of children and adults. J Fam Violence 18:107–120 10.1023/A:1022841215113

[8] Herrenkohl TI, Hong S, Klika JB, Herrenkohl RC, Russo MJ (2013) Developmental impacts of child abuse and neglect related to adult mental health, substance use, and physical health. J Fam Violence 28:191–199 10.1007/s10896-012-9474-9 PMC3839858 24285915

[9] Huh HJ, Kim SY, Yu JJ, Chae JH (2014) Childhood trauma and adult interpersonal relationship problems in patients with depression and anxiety disorders. Ann Gen Psychiatry 13:1–13 25648979 10.1186/s12991-014-0026-y PMC4304140

[10] Mendoza MM, Dmitrieva J, Perreira KM, Hurwich-Reiss E, Watamura SE (2017) The effects of economic and sociocultural stressors on the well-being of children of latino immigrants living in poverty. Cultur Divers Ethnic Minor Psychol 23:15–26 28045307 10.1037/cdp0000111 PMC5338689

[11] Lopez MH, Gonzalez-Barrera A, Krogstad JM(2018) *Latinos and discrimination*.Pew Research Center. https://www.pewresearch.org/hispanic/2018/10/25/

[12] Drake B, Jolley JM, Lanier P, Fluke J, Barth RP, Johnson-Reid M (2011) Racial bias in child protection? A comparison of competing explanations using national data. Pediatrics 127:471–478 21300678 10.1542/peds.2010-1710 PMC9923773

[13] Fluke J, Harden BJ, Jenkins M, Ruehrdanz A(2010) Research synthesis on child welfare: Disproportionality and disparities. The Center for the Study of Social Policy and The Annie E. Casey Foundation on behalf of The Alliance for Racial Equity in Child Welfare, Washington, DC

[14] Lambert JE, Holzer J, Hasbun A (2014) Association between parents’ PTSD severity and children’s psychological distress: a meta-analysis. J Trauma Stress 27:9–17 24464491 10.1002/jts.21891

[15] Milner JS, Thomsen CJ, Crouch JL, Rabenhorst MM, Martens PM, Dyslin CW et al (2010) Do trauma symptoms mediate the relationship between childhood physical abuse and adult child abuse risk? Child Abuse Negl 34:332–344 20359748 10.1016/j.chiabu.2009.09.017

[16] Finzi-Dottan R, Harel G (2014) Parents’ potential for child abuse: an intergenerational perspective. J Fam Violence 29:397–408 10.1007/s10896-014-9592-7

[17] Borelli JL, Cohen C, Pettit C, Normandin L, Target M, Fonagy P et al (2019) Maternal and child sexual abuse history: an intergenerational exploration of children’s adjustment and maternal trauma-reflective functioning. Front Psychol 10:1062 31156503 10.3389/fpsyg.2019.01062 PMC6530340

[18] Russotti J, Warmingham JM, Handley ED, Rogosch FA, Cicchetti D (2021) Child maltreatment: an intergenerational cascades model of risk processes potentiating child psychopathology. Child Abuse Negl 112:104829 33359770 10.1016/j.chiabu.2020.104829 PMC7855935

[19] Cerdeña JP, Rivera LM, Spak JM (2021) Intergenerational trauma in Latinxs: a scoping review. Soc Sci Med 270:113662 33476987 10.1016/j.socscimed.2020.113662

[20] Briggs RD, Silver EJ, Krug LM, Mason ZS, Schrag RDA, Chinitz S et al (2014) Healthy steps as a moderator: the impact of maternal trauma on child social-emotional development. Clin Pract Pediatr Psychol 2:166–175

[21] McFarlane J, Fredland NM, Symes L, Zhou W, Jouriles EN, Dutton MA et al (2017) The intergenerational impact of intimate partner violence against mothers on child functioning over four years. J Fam Violence 32:645–655 10.1007/s10896-017-9913-8

[22] Keyes KM, Gary D, O’Malley PM, Hamilton A, Schulenberg J (2019) Recent increases in depressive symptoms among US adolescents: trends from 1991 to 2018. Soc Psychiatry Psychiatr Epidemiol 54:987–996 30929042 10.1007/s00127-019-01697-8 PMC7015269

[23] Robinson BA, Hendrix CL, Sloan Krakovsky H, Smith AK, Brennan PA (2019) Maternal trauma exposure and childhood anxiety outcomes: examining psychosocial mechanisms of risk. J Abnorm Child Psychol 47:645–657 30112594 10.1007/s10802-018-0463-1 PMC9484555

[24] Abrams KY, Rifkin A, Hesse E (2006) Examining the role of parental frightened/frightening subtypes in predicting disorganized attachment within a brief observational procedure. Dev Psychopathol 18:345–361 16600058 10.1017/S0954579406060184

[25] Hesse E, Main M (2006) Frightened, threatening, and dissociative parental behavior in low-risk samples: description, discussion, and interpretations. Dev Psychopathol 18:309–343 16600057 10.1017/S0954579406060172

[26] Jacobvitz D, Leon K, Hazen N (2006) Does expectant mothers’ unresolved trauma predict frightened/frightening maternal behavior? Risk and protective factors. Dev Psychopathol 18:363–379 16600059 10.1017/S0954579406060196

[27] Samuelson KW, Wilson CK, Padrón E, Lee S, Gavron L (2017) Maternal PTSD and children’s adjustment: parenting stress and emotional availability as proposed mediators. J Clin Psychol 73:693–706 27487248 10.1002/jclp.22369

[28] Schwerdtfeger KL, Goff BSN (2007) Intergenerational transmission of trauma: exploring mother-infant prenatal attachment. J Trauma Stress 20:39–51 17345647 10.1002/jts.20179

[29] Copeland WE, Shanahan L, Hinsesley J, Chan RF, Aberg KA, Fairbank JA, van den Oord EJCG, Costello EJ (2018) Association of childhood trauma exposure with adult psychiatric disorders and functional outcomes. JAMA Netw 1:e184493 10.1001/jamanetworkopen.2018.4493 PMC6324370 30646356

[30] Cicchetti D, Cohen DJ (2006) Developmental psychopathology: risk, disorder, and adaptation, 2nd edn. John Wiley & Sons, Inc.

[31] Karlsen S, Nazroo JY (2002) Relation between racial discrimination, social class, and health among ethnic minority groups. Am J Public Health 92:624–631 11919063 10.2105/AJPH.92.4.624 PMC1447128

[32] Ayón C, Marsiglia FF, Bermudez-Parsai M (2010) Latino family mental health: exploring the role of discrimination and familismo. J Community Psychol 38:742–756 20890371 10.1002/jcop.20392 PMC2947026

[33] Moradi B, Risco C (2006) Perceived discrimination experiences and mental health of Latina/o American persons. J Couns Psychol 53:411–421 10.1037/0022-0167.53.4.411

[34] Sanders-Phillps K, Settles-Reaves B, Walker D, Brownlow J (2009) Social inequality and racial discrimination: risk factors for health disparities in children of color. Pediatrics 124:S176–S186 19861468 10.1542/peds.2009-1100E

[35] Comas-Díaz L, Hall GN, Neville HA (2019) Racial trauma: theory, research, and healing: introduction to the special issue. Am Psychol 74:1–5 30652895 10.1037/amp0000442

[36] Williams DR, Neighbors HW, Jackson JS (2003) Racial/ethnic discrimination and health: findings from community studies. Am J Public Health 93:200–208 12554570 10.2105/AJPH.93.2.200 PMC1447717

[37] Huynh VW, Fuligni AJ (2010) Discrimination hurts: the academic, psychological, and physical well-being of adolescents. J Adolesc Res 20:916–941 10.1111/j.1532-7795.2010.00670.x

[38] Prelow HM, Danoff-Burg S, Swenson RR, Pulgiano D (2004) The impact of ecological risk and perceived discrimination on the psychological adjustment of african american and european american youth. J Community Psychol 32:375–389 10.1002/jcop.20007

[39] Carter RT, Forsyth J (2010) Reactions to racial discrimination: emotional stress and help-seeking behaviors. Psychol Trauma 2:183–191 10.1037/a0020102

[40] Mekawi Y, Carter S, Brown B, Martinez de Andino A, Fani N, Michopoulos V et al (2021) Interpersonal trauma and posttraumatic stress disorder among Black Women: does racial discrimination matter? J Trauma Dissociation 22:154–169 33460354 10.1080/15299732.2020.1869098 PMC9082823

[41] Cicchetti D, Lynch M (1993) Toward an ecological/transactional model of community violence and child maltreatment: consequences for children’s development. Psychiatry 56:96–118 8488217 10.1080/00332747.1993.11024624

[42] Delgado MY, Nair RL, Zeiders KH, Jones SK (2019) Latino adolescents’ experiences with ethnic discrimination: moderating factors and mediating mechanisms. Handbook of children and prejudice. Springer, Cham, pp 515–531

[43] Lawson DM, Quinn J (2013) Complex trauma in children and adolescents: evidence-based practice in clinical settings. J Clin Psychol 69:497–509 23564579 10.1002/jclp.21990

[44] Stevens JS, van Rooi SJ, Jovanovic T (2016) Developmental contributors to trauma response: the importance of sensitive periods, early environment, and sex differences. Beh Neuro of PTSD 1:1–22 10.1007/7854_2016_38 PMC5425320 27830573

[45] Evans SE, Davies C, DiLillo D (2008) Exposure to domestic violence: a meta-analysis of child and adolescent outcomes. Aggress Violent Behav 13:131–140 10.1016/j.avb.2008.02.005

[46] Andrews AR, Jobe-Shields L, López CM, Metzger IW, de Arellano MA, Saunders B et al (2015) Polyvictimization, income, and ethnic differences in trauma-related mental health during adolescence. Soc Psychiatry Psychiatr Epidemiol 50:1223–1234 26048339 10.1007/s00127-015-1077-3 PMC4521986

[47] López CM, Andrews AR, Chisolm AM, de Arellano MA, Saunders B, Kilpatrick DG (2017) Racial/ethnic differences in trauma exposure and mental health disorders in adolescents. Cultur Divers Ethnic Minor Psychol 23:382–287 27786496 10.1037/cdp0000126 PMC5408300

[49] Borelli J, Yates T, Hecht H, Cervantes B, Russo L, Arreola J et al (2020) Confía en mí, Confío en ti: applying developmental theory to mitigate sociocultural risk in latinx families. Dev Psychopathol 33:581–597 10.1017/S0954579420001364 PMC8105258 33269671

[49] Kubany ES, Haynes SN, Leisen MB, Owens JA, Kaplan AS, Watson SB et al (2000) Development and preliminary validation of a brief broad-spectrum measure of trauma exposure: the traumatic life events Questionnaire. Psychol Assess 12:210–224 10887767 10.1037/1040-3590.12.2.210

[50] Himmerich SJ, Seligowski AV, Orcutt HK (2019) The impact of child abuse on relationships between resource loss and posttraumatic stress: a cross-lagged panel analysis. J Trauma Dissociation 20:619–633 30932781 10.1080/15299732.2019.1597811

[51] Finch BK, Kolody B, Vega WA (2000) Perceived discrimination and depression among mexican-origin adults in CA. J Health Soc Behav 41:295–313 11011506 10.2307/2676322

[52] Ornelas IJ, Perreira KM (2011) The role of migration in the development of depressive symptoms among latino immigrant parents in the USA. Soc Sci Med 8:1169–1177 10.1016/j.socscimed.2011.07.002 PMC3185160 21908089

[53] Borelli JL, Russo LN, Arreola J, Cervantes BR, Hecht HK, Leal F et al (2021) Más fuertes juntos: attachment relationship quality, but not demographic risk, predicts psychopathology in Latinx. J Community Psychol 49:2086–2105 33635588 10.1002/jcop.22535

[54] March JS, Parker JDA, Sullivan K, Stallings P, Conners C (1997) The multidimensional anxiety scale for children (MASC): factor structure, reliability, and validity. J Am Acad Child Adolesc Psychiatry 36:554–565 9100431 10.1097/00004583-199704000-00019

[55] Martinez W, Polo AJ, Carter JS (2012) Family orientation, language, and anxiety among low-income latino youth. J Anxiety Disord 26:517–525 22410091 10.1016/j.janxdis.2012.02.005

[56] Achenbach TM (1991) Manual for the child behavior checklist/4–18 and 1991 profile. University of Vermont, Department of Psychiatry

[57] Polo AJ, Lopez SR (2009) Culture, context, and the internalizing distress of mexican american youth. J Clin Child Psychol 38:273–285 10.1080/15374410802698370 19283605

[58] Kovacs M (1992) Children’s depression inventory, manual. MultiHealth Systems

[59] McNaughton DB, Cowell JM, Gross D, Fogg L, Ailey SH (2004) The relationship between maternal and child mental health in mexican immigrant families. Res Theory Nurs Pract 18:229–242 15553349 10.1891/rtnp.18.2.229.61283

[60] Derogatis LR (2001) Brief symptom inventory (BSI)-18: administration, scoring and procedures manual. NCS Pearson

[61] Prelow HM, Weaver SR, Swenson RR, Bowman MA (2005) A preliminary investigation of the validity and reliability of the brief-symptom Inventory‐18 in economically disadvantaged Latina American mothers. J Community Psychol 33:139–155 10.1002/jcop.20041

[62] Hooper LM, Stockton P, Krupnick JL, Green BL (2011) Development, use, and psychometric properties of the Trauma History Questionnaire. J Loss Trauma 16:258–283 10.1080/15325024.2011.572035

[63] Garcia-Campayo J, Navarro-Gil M, Andrés E, Montero-Marin J, Lopez-Artalm L et al (2014) Validation of the spanish versions of the long (26 items) and short (12 items) forms of the Self-Compassion Scale (SCS). Health Qual Life Outcomes 12:4 24410742 10.1186/1477-7525-12-4 PMC3896764

[64] Cervantes RC, Fisher DG, Padilla AM, Napper LE (2016) The hispanic stress Inventory Version 2: improving the assessment of acculturation stress. Psychol Assess 28:509–522 26348029 10.1037/pas0000200 PMC4781681

[65] Alda M, Minguez J, Montero-Marin J, Gili M, Puebla-Guedea M, Herrera-Mercadal et al(2015) Validation of the spanish version of the Multidimensional State Boredom Scale (MSBS). Health Qual Life Outcomes 13. 10.1186/s12955-015-0252-2 PMC4443509 25975274

[66] Briggs-Gowan MJ, Carter AS, Clark R, Augustyn M, McCarthy KJ, Ford JD (2010) Exposure to potentially traumatic events in early childhood: differential links to emergent psychopathology. J Child Psycho Psychiatry 51:1132–1140 10.1111/j.1469-7610.2010.02256.x PMC3106304 20840502

[67] Plant DT, Pawlby S, Pariante CM, Jones FW (2018) When one childhood meets another–maternal childhood trauma and offspring child psychopathology: a systematic review. Clin Child Psychol Psychiatry 23:483–500 29171287 10.1177/1359104517742186

[68] Hayes AF (2012) “PROCESS: a Versatile Computational Tool for observed variable mediation, moderation, and conditional process modeling,” white paper. The Ohio State University

[69] Colby SL, Ortman JM(2015) Projections of the size and composition of the US population: 2014 to 2060. Retrieved from U.S. Census Bureau website: https://www.census.gov/content/Census/library/publications/2015/demo/p25-1143.pdf

[70] Luken A, Nair R, Fix RL (2021) On racial disparities in child abuse reports: exploratory mapping the 2018 NCANDS. Child Malt 1:1–15 10.1177/10775595211001926 PMC8328863 33729016

[71] National Healthcare Quality and Disparities (NHQD) Report (2019) Agency for Healthcare Research and Quality. AHRQ Publication No. 19-0070‐EF. Rockville, MD

[72] Murry VM, Brown PA, Brody GH, Cutrona CE, Simons RL (2001) Racial discrimination as a moderator of the links among stress, maternal psychological functioning, and family relationships. J Marriage Fam 63:915–926 10.1111/j.1741-3737.2001.00915.x

[73] Moody G, Cannings-John R, Hood K, Kemp A, Robling M (2018) Establishing the international prevalence of self-reported child maltreatment: a systematic review of maltreatment type and gender. BMC Pub Healt 18:1164 10.1186/s12889-018-6044-y PMC6180456 30305071

[74] Clemmons JC, DiLillo D, Martinez IG, DeGue S, Jeffcott M (2003) Co-occuring forms of child-maltreatment and adult adjustment reported by Latina college students. Child Abuse Negl 27:751–767 14627077 10.1016/S0145-2134(03)00112-1

[75] Pina AA, Gonzales NA, Mazza GL, Gunn HJ, Holly LE, Stoll RD et al (2020) Streamlined prevention and early intervention for pediatric anxiety disorders: a randomized controlled trial. Prev Sci 21:487–497 31927654 10.1007/s11121-019-01066-6 PMC7166170

[76] Ramos G, Ponting C, Bocanegra E, Chodzen G, Delgadillo D, Rapp A et al (2021) Discrimination and internalizing symptoms in rural latinx adolescents: the protective role of family resilience. J Clin Child Adolesc Psychol 1:1–14 10.1080/15374416.2021.1923018 34038290

[77] Cooley-Quille M, Boyd R, Frantz E, Walsh J (2001) Emotional and behavioral impact of exposure to community violence in inner‐city adolescents. J Clin Child Adolesc Psychol 30:199–206 10.1207/S15374424JCCP3002_7 11393920

